# Development of a fixed list of descriptors for the qualitative behavioral assessment of thoroughbred horses in the racing environment

**DOI:** 10.3389/fvets.2023.1189846

**Published:** 2023-08-10

**Authors:** Fernando Mosquera Jaramillo, Tiago Marcelo Oliveira, Pedro Enrique Ayres Silva, Pedro Henrique Esteves Trindade, Raquel Yvonne Arantes Baccarin

**Affiliations:** ^1^Department of Internal Medicine, School of Veterinary Medicine and Animal Science, University of São Paulo, São Paulo, Brazil; ^2^Equine Centre, Melbourne Veterinary School, Faculty of Science, University of Melbourne, Werribee, VIC, Australia; ^3^Graduate Program in Anesthesiology, Medical School, São Paulo State University (Unesp), Botucatu, Brazil; ^4^Department of Population Health and Pathobiology, College of Veterinary Medicine, North Carolina State University (NCSU), Raleigh, NC, United States

**Keywords:** racehorses, behavior, thoroughbred, environment, QBA

## Abstract

**Introduction:**

Horse racing is a major sport practiced worldwide. The environment to which horses are exposed during race meetings can influence their behavior. However, to the best of our knowledge, a method for assessing a horse’s response to its surroundings during the pre- and post-race periods has not yet been reported. This study aimed to create a standard list of descriptors for use in a qualitative behavioral assessment (QBA) focused on assessing the emotional expressivity of horses before and after racing events.

**Materials and methods:**

Seventy pre- or post-race 30-second videos of horses were randomly selected from our database of 700 videos. A panel of 8 experienced equine sports medicine specialist veterinarians watched a 60 min presentation on QBA. The panel then watched all videos randomly, simultaneously, individually, continuously, and without any verbal interaction, describing the descriptors related to the emotional expressivity of the horse after each video using a method known as free-choice profiling (FCP).

**Results:**

The initial selection of descriptors was based on those indicated by more than one evaluator in the same video, or descriptors with more than 20 occurrences. The second selection was performed based on the content validity index (CVR) to select the descriptors retained in the previous step. Another panel of six veterinarians scored each of the descriptors retained for content validity on a visual scale. Interobserver reliability was estimated using the intraclass correlation coefficient (ICC) and its respective 95% confidence intervals (CI). A natural language processing (NLP) algorithm was used to analyze the behavior (positive or negative polarity) of the descriptors based on the lexicoPT package of R software.

**Discussion/Conclusion:**

NLP analysis considered the descriptors “agitated,” “troubled,” “restless” and “irritated” to have a negative polarity, while “focused,” “relaxed” and “peaceful” had a positive polarity. In the principal component analysis (PCA), descriptors in a negative state were associated with each other and inversely associated with descriptors in a positive state. We conclude with a fixed list of descriptors to be used in a QBA to assess emotional and welfare expressivity in racehorses’ pre- and post-race environments.

## 1. Introduction

Horse racing is a major sport practiced worldwide (1, 2). Immediately before participating in a race, horses are led from their stalls to a common yard where they are paraded and mounted before entering the track. Although little is know about this pre-race environment, it may influence athletic performance and behavior of participating horses (3). For a horse to achieve optimal performance, in addition to being in its best physical and mental condition, it must also be in an excellent state of welfare (4, 5). However, it is a big challenge to describe and classify behaviors that express emotional states in this environment (3).

Qualitative behavior assessment (QBA) is a scientific method of evaluating animal emotional expressions in different situations. This method uses descriptors of behavior that can be classified as positive (e.g., relaxed and curious), negative (e.g., tense and irritating), and neutral (e.g., active and attentive), in which an evaluator scores the degree of expression of each descriptor displayed by the animal on a visual analog scale after a short period of observation (6, 7). This contrasts with the occurrence or recording of the duration of behaviors, which are quantitative methods.

QBA requires a list of specific descriptors for each environment, situation, and target species, and there are few papers that applied this method in horses (8, 9). Descriptors can originate from a fixed list common to all evaluators, or from individual lists created by each evaluator. These private lists can be created by a method known as free-choice profiling (FCP), in which each evaluator suggests their own descriptors with reference to their personal experience after observing the animal’s emotional expressivity in different sports and natural situations. The descriptors of a fixed list can be determined theoretically or established empirically based on a mining of the descriptors obtained by an FCP conducted by different evaluators (8–11).

This qualitative method proved to be suitable for studying responsiveness to environmental challenges (represented by an open field test) in horses and ponies (12), classifying behaviors as standing still, approaching, sniffing, nibbling clothes, nibbling hay, vocalizing, sniffing the environment, and moving away during interactions with known and unknown humans (13). Recently, QBA has been used to identify human-animal behaviors in dairy calves and their handlers (14). The QBA proved to be an adequate tool for identifying the emotional expressivity of donkeys (15), the behavior of pigs when they won or lost a social dispute (16), and the emotional expression of dogs in shelters (11, 17). In horses, QBA is applied during the veterinary check in an endurance event, when the horses had completed a stage of the exercise. According to the authors, this is a reliable, noninvasive, and rapid method for evaluating the emotional expressivity of horses. When combined with evidence from physical examinations, much information was obtained about the condition of the horses, which is useful for assessing the probable performance of horses in different disciplines and reflects states (e.g., fatigue) that are difficult to assess using other methods (9).

It is possible that the QBA is useful for evaluating the behavior of Thoroughbred horses in the race environment. Therefore, this study aimed to create a fixed list of descriptors for use in a QBA that focused on assessing the emotional expressivity of horses during racing events. Our hypothesis is that horses in this environment express behaviors that can be captured by descriptors generated in a systematic and standardized way.

## 2. Materials and methods

### 2.1. Animals and video recording

This study was approved by the Committee on Ethics in Animal Use (# 8656240521) of the School of Veterinary Medicine and Animal Science, University of São Paulo, Brazil. Seven hundred 30 s videos of Thoroughbred horses (of both sexes) presented for racing from September to December 2021 at the Jockey Club of São Paulo in Brazil were collected. These 30 s videos [time adapted from Napolitano et al. (12)] were collected with the horse being filmed from a fixed point approximately on meter away in front of the horses’head, and it was possible to observe the entire body of each horse during the pre-race assessment (approximately an hour before the race) or after the race. Pre-race evaluation was routinely performed by veterinarians from the Department of Veterinary Assistance (DVA) for all participating horses and consisted of animal identification and physical evaluation of suitability for racing. Seventy randomly selected videos were used in this study, 63 videos of pre-race horses and seven of post-race horses in the same place, and each horse was evaluated only once.

### 2.2. Free choice profiling

A panel of eight veterinarians with more than 10 years in equine sports medicine and race environment but no previous experience with FCP or QBA attended a 60 min presentation on QBA. Soon after, the committee watched 70 videos individually, both simultaneously and in a random sequence. There was no verbal interaction between panel members, and this process generated a list of descriptors established by free-choice profiling (FCP). For this, after a 30 s video display, each panel member had up to 1 min to write down descriptors for the body and facial language displayed in the recording according to their experience. The complete evaluation lasted for 110 min. Descriptors were selected from the Brazilian Portuguese language (Supplementary Table S1).

### 2.3. Content validity

To validate the content of the fixed list, 10 veterinary specialists who worked with horses, none of whom participated in the FCP, were consulted regarding descriptors recorded by more than one evaluator in the same video or descriptors with more than 20 occurrences in the FCP. The specialists responded to five questions using a Likert scale (with grade 1 corresponding to “not at all” and grade 5 corresponding to “completely”). The questions evaluated the descriptors in relation to understanding and intuition, ambiguity, usefulness in describing the racing environment, and relevance in assessing the welfare and physical performance of horses in the racing environment (Table 1).

**Table 1:** Description of the five questions answered by the evaluators using a Likert scale.

Questions	Scale
Easy understanding and intuition?	1□2 □3 □4 □5 □
Ambiguous, can it be interpreted with different meanings?	1□2 □3 □4 □5 □
Useful for describing emotions in a race event environment?	1□2 □3 □4 □5 □
Relevant to assess the welfare of horses in the racing environment?	1□2 □3 □4 □5 □
Relevant to assess horse performance in turf?	1□2 □3 □4 □5 □

### 2.4. Visual analog scale

A third panel of six veterinarians specializing in sporting horses who did not participate in the two previous stages watched 70 videos randomly, simultaneously, individually, continuously, without any verbal interactions. After each 30 s video display, each expert had up to 1 min to score on an analog scale how much the animals displayed the descriptors listed in the previous step. The evaluations were all performed on the same day with an interval of 15 min after the thirty-fifth video, the complete evaluation lasted 120 min.

### 2.5. Statistical description

All statistical analyses were performed by a data scientist (PHET) in R software with the integrated development environment RStudio [Version 4.1.0 (2021-06-29), RStudio, Inc.]. The functions and packages used were presented in the format “package::function” corresponding to the programming language in R. For all tests, a level of 5% was considered significant. All figures were constructed using a palette suitable for colorblind people (ggplot2::scale_ color _viridis_d).

The initial selection of descriptors was based on those with more than 20 observations, or descriptors indicated by more than one observer for the same video during the FPC.

The second selection was conducted using the descriptors retained in the descriptive stage for content validity. To this end, the content validity index CVR [adapted from Streiner and Norman (18)] was calculated by applying the following equation to each of the five questions used for content validity:

CVR=ne−N2N2

Where “*n_e_*” is the number of raters who considered the descriptor essential (a rating of 3, 4 or 5 for Q1, Q3, Q4 and Q5, as well as a rating of 1, 2 or 3 for Q2) and “*N*” is the total number of raters.

The CVR ranges from −1 to +1. To ensure that the results are not due to chance, a CVR value >0.62 is recommended to interpret the descriptor as essential in each question, in a scenario with 10 raters (19). In this step, the descriptor was retained when it had a CVR value >0.62 for at least three out of five questions.

The reliability of the eight observers, together with the descriptors retained by the CVR, was estimated by applying the intraclass correlation coefficient (ICC) with a bidirectional random effects model using the multirater agreement type and its 95% confidence interval (CI) (irr::icc). Only descriptors with an ICC CI >0.60 were retained in this step.

A natural language processing (NLP) algorithm was used to analyze the sentiment (positive or negative polarity) of the descriptors (lexicon PT:get_word_sentiment).

Finally, principal component analysis (PCA; stats::princomp) was conducted with the descriptors retained in the reliability step to analyze multiple associations between them. Because the purpose of PCA is to reduce dimensionality, Horn’s parallel analysis (psych::fa.parallel) was conducted to select the optimal number of principal components (PC) to be analyzed among the PCs originating from the PCA. A biplot containing the descriptors retained by reliability was constructed, and the observations were colored according to the observers for a qualitative analysis of their distribution.

## 3. Results

### Free choice profiling

**Figure 1:**
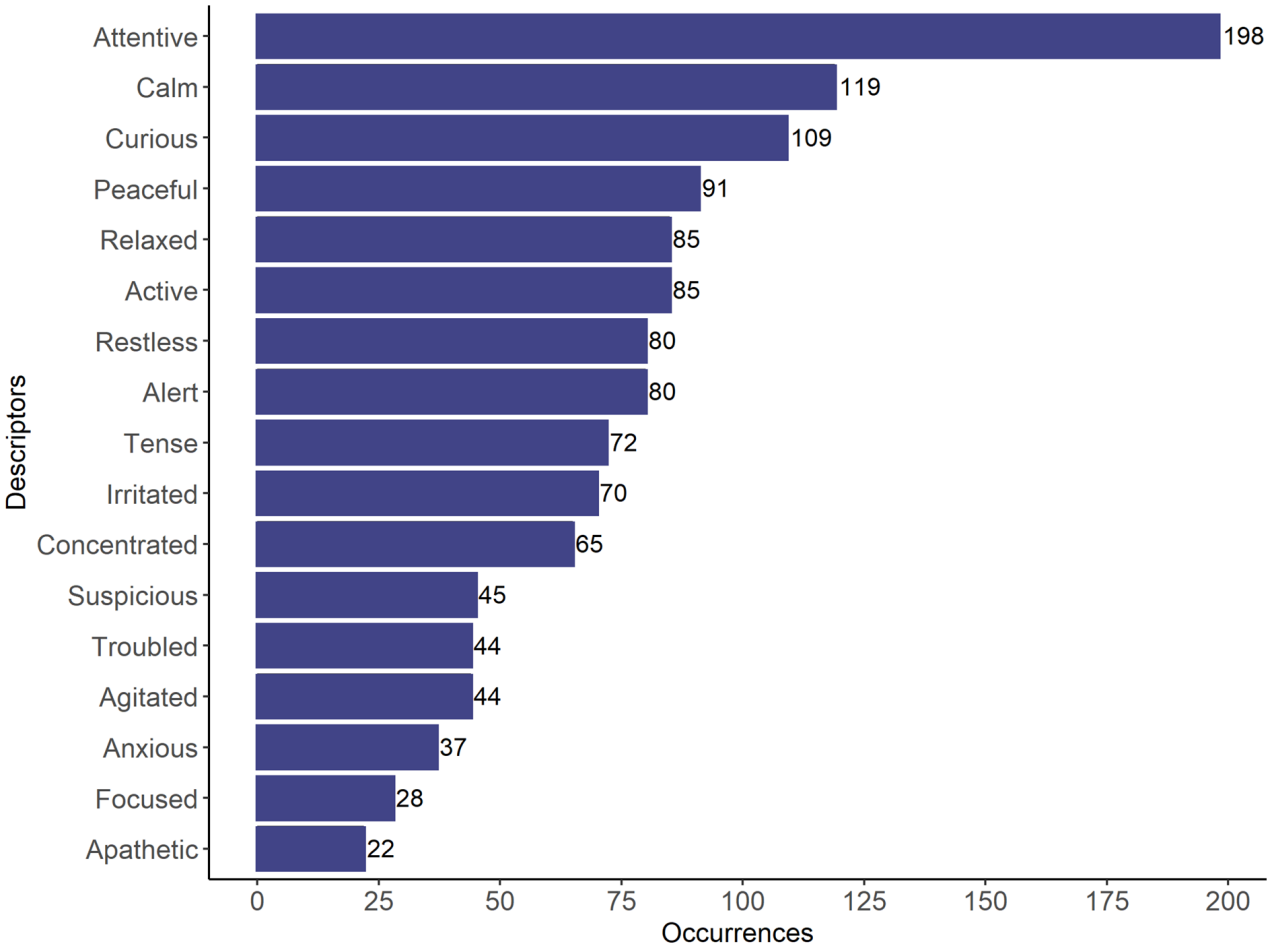
Bar graph showing the 17 descriptors that were indicated by more than one rater in the same video.

The FCP generated 200 different descriptors, of which 16 descriptors had more than 20 occurrences (attentive, curious, calm, concentrated, relaxed, anxious, tense, bothered, peaceful, restless, suspicious, alert, active, irritated, agitated and focused) or were indicated by more than one observer in the same video (attentive, curious, calm, concentrated, relaxed, anxious, tense, troubled, peaceful, restless, suspicious, alert, active, irritated, agitated, focused, and apathetic; Figure 1), this step retained 17 descriptors.

### Content validity

Eleven descriptors were retained for content validity, showing a CVR >0.62 in at least three of the five questions asked (Table 2).

**Table 2:** The descriptors shown in bold were approved in at least three questions; in gray are descriptons with a CVR > 0.62.

Descriptors	Qst-1	Qst-2	Qst-3	Qst-4	Qst-5
**Agitate**	1	0.6	1	0.8	0.2
**Alert**	1	1	0.8	0.8	0.8
**Apathetic**	1	1	0.8	1	1
**Attentive**	1	1	1	0.6	0.8
Active	0.6	0.2	0.8	0.8	0.6
Calm	0.8	0.2	0.6	0.6	0.4
Concentrated	0.6	0.4	0.8	0.8	0.6
Curious	0.8	0.6	0.4	0.4	−0.2
Suspicious	0	0.2	0.4	0.6	−0.2
**Focused**	0.6	0.8	0.6	1	0.8
**Troubled**	1	1	1	0.8	0.4
**Restless**	1	0.8	0.6	0.8	0.2
**Irritated**	1	0.4	1	1	0.8
**Relaxed**	1	0.6	0.8	0.8	0.6
**Tense**	1	0.6	1	0.8	0.6
**Peaceful**	1	0.8	0.8	0.6	0.4

### Visual analog scale

**Figure 2:**
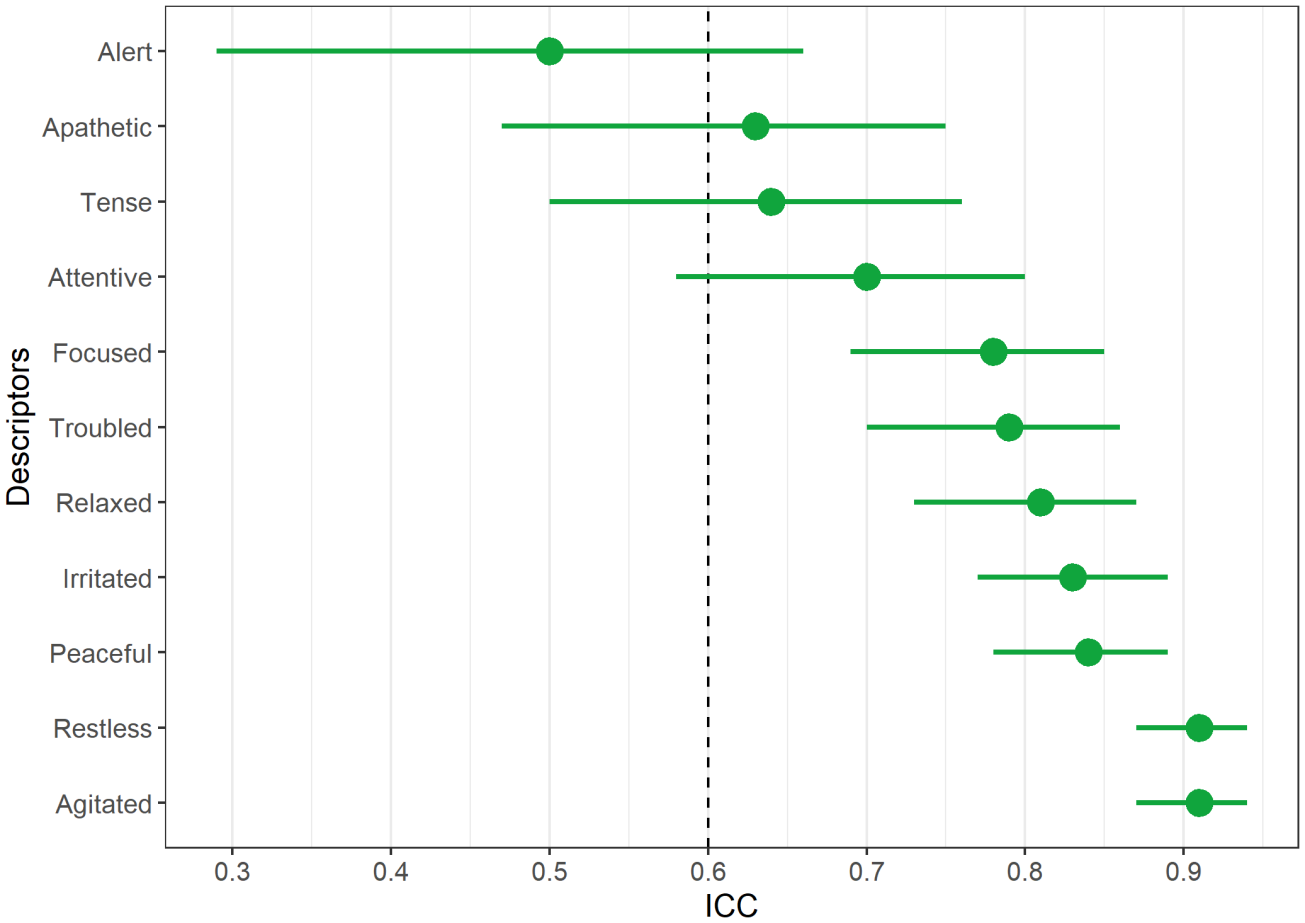
Showing the ICC and 95% confidence interval of the visual analog scale.

On the visual analog scale, seven descriptors had an ICC >0.70 and a low confidence interval (focused, troubled, relaxed, irritating, peaceful, restless, and agitated), and were therefore retained in this analysis (Figure 2).

**Figure 3:**
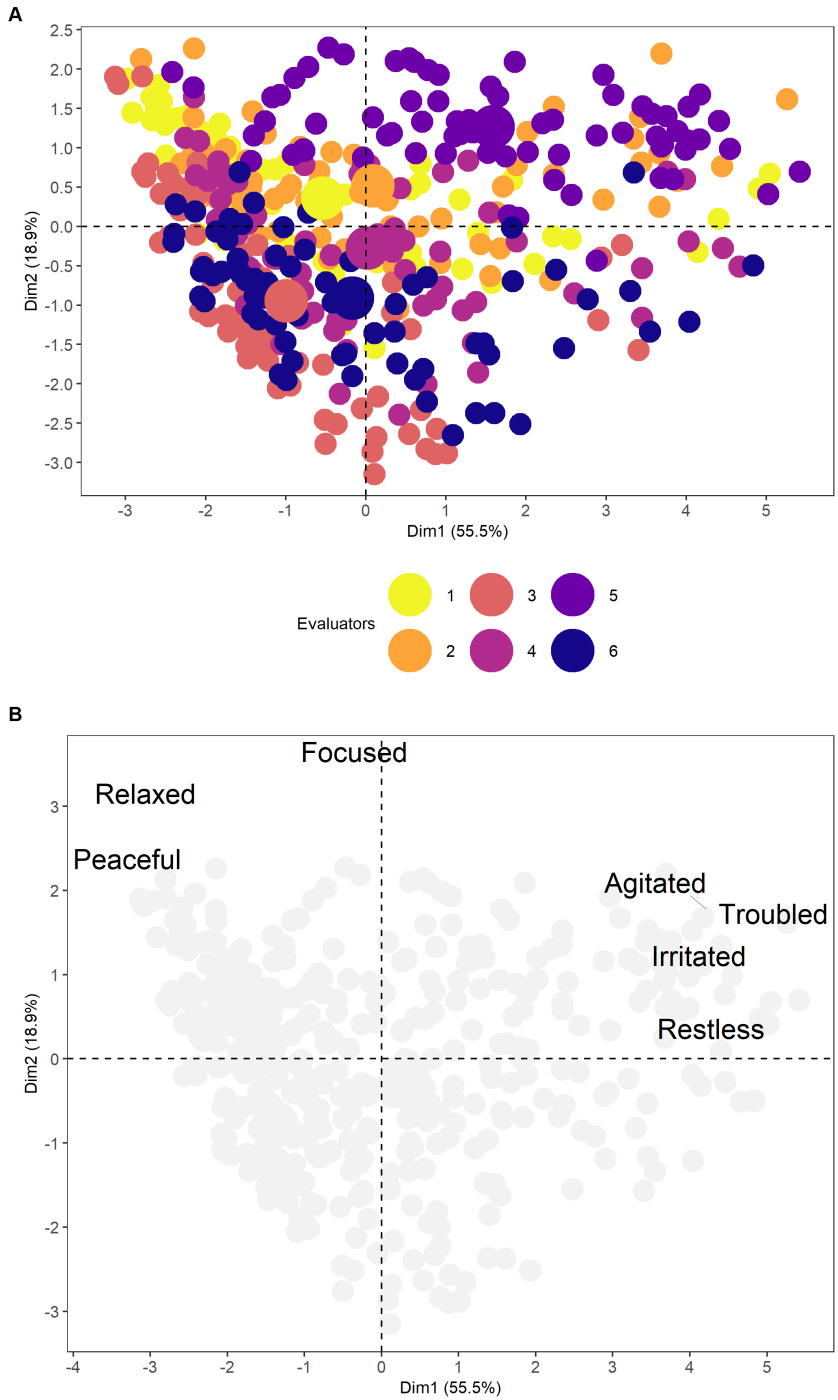
**(A)** The biplot shows the similarity of the six evaluators. **(B)** It shows the separation of descriptors according to polarity, therefore, agitated, troubled, restless, and irritated as negative; and focused, relaxed, and peaceful as a positive.

Horne’s parallel analysis indicated the optimal number of two principal components (CP) to be analyzed in the PCA (Table 3). CP 1 and 2 together explained 74.41% of the total variance in the data. Agitated, troubled, restless, and irritable showed a positive association with CP 1, while relaxed and peaceful showed a negative association with CP 1. Focused, relaxed, and peaceful activities are positively association with CP 2. Although focused alone showed a correlation with CP 3 it was not analyzed because of the presence of two CP according to Horn’s parallel analysis. Such associations show a segregation of the polarities of each descriptor and the CP could be named as “negative polarity” and “positive polarity” respectively for CP 1 and 2. The biplot illustrates the similarity of the raters (Figure 3A) and the separation of the descriptors according to the polarity found by sentiment analysis of the NLP, which was agitated, troubled, restless, and irritating as negative and focused, relaxed, and peaceful as positive (Figure 3B). In the PCA, descriptors with negative sentiments were associated with each other and inversely associated with descriptors with positive sentiments.

**Table 3:** Load values, eigenvalues, and principal component analysis (PCA) variance.

Descriptors	CP 1^*^	CP 2^*^	CP 3	CP 4	CP 5	CP 6	CP 7
Agitated	**0.82**	0.34	0.06	0.40	0.02	0.17	0.15
Focused	−0.22	**0.68**	**−0.69**	0.02	−0.06	−0.01	−0.08
Troubled	**0.87**	0.29	−0.02	−0.27	−0.02	−0.19	0.21
Restless	**0.88**	0.11	0.23	0.10	−0.32	−0.09	−0.19
Irritated	**0.85**	0.28	0.12	−0.25	0.28	0.13	−0.16
Relaxed	**−0.65**	**0.57**	0.39	0.19	0.18	−0.19	−0.04
Peaceful	**−0.70**	**0.50**	0.35	−0.24	-0.21	0.17	0.06
Eigenvalue	3.88	1.33	0.83	0.40	0.26	0.15	0.14
Variance (%)	55.48	18.93	11.82	5.78	3.73	2.21	2.06
Accumulated variance (%)	55.48	74.41	86.23	92.01	95.74	97.94	100.00

CP, principal component; * indicates the indicated CP to be retained by Horn’s parallel analysis; in bold are loading values > 0.40 or or < −0.40.

## 4. Discussion

The results of this study, carried out with horses in a pre-and post-race environment, yielded very similar descriptors to earlier work in different situations such as an endurance environment (9), where the authors evaluated the emotional state of the horses before, during and at the end of a 160 km endurance event. In another study (8) of emotional expressivity evaluations of horses submitted to short-term treatments, they showed emotional expressivity similar to those found with our descriptors regarding valence and arousal, and in studies of dogs in shelter environments (11) and dogs in a home environment (20).

The polarity results were similar to those obtained in a study conducted with horses exposed to two positive and two negative expressivity situations. The horses were filmed for 60 s for each treatment response. Eighty videos of 45 s were evaluated by 15 veterinary students with different levels of experience with horses, allowing the association of positive terms (calm/relaxed/content) with positive situations, and negative terms (stressed/nervous) with negative situations (8). In our study we analyzed 30 s videos, also generating descriptors with positive, negative e neutral polarity. However, a fixed binary application of certain terms may be inaccurate for physical performance and welfare assessments. For example an animal classified as “relaxed” may reflect something of a positive (relaxation) or negative (tiredness) polarity, similarly “focused” can reflect something of a positive (concentrated) or negative (worried) polarity.

In a study carried out with foals, the authors filmed and evaluated the emotional expressivity response when the animals had contact with an unknown person and were able to correlate negative responses such as distrustful, nervous, restless, worried, tense, before handling by an unknown person, and positive responses such as explorer, sociable, calm, relaxed, safe, after handled by an unknown person (13). In another study, ponies were placed in an environment that differed from their usual one. The behavior was recorded, and the evaluators analyzed 20 video clips of 2.5 min duration. Terms that summarized the response of each animal to the test situation were recorded to obtain descriptors with similar polarity to those in our study (12). The descriptors obtained in a study of shelter dogs also generated a polarity classification in which 13 observers evaluated 16 video clips of shelter dogs in different scenarios (individual/pair/group housing and presence/absence of human activity). Both positive polarity correlation (playful, sociable, curious, happy, and interested) and negative polarity correlation (uncomfortable, bored, apathetic, anxious, stressed, and depressed) (11).

The study, carried out on sports endurance horses, used 22 evaluators, of which 14 were classified as experienced with horses. After evaluating each of the 16 videos for 45–70 s, the evaluators had up to 2 min to write the terms they considered most adequate to describe the qualities expressed by the animals (9). Some of the descriptors obtained agreed with our descriptors (calm/content/relaxed and agitated/angry/annotated) and with their classifications. The authors of that study did not include the descriptor “alert” as it was considered ambiguous, which was the same reason for its removal from our list after the evaluation stage. This may have occurred because both studies were conducted in a sporting environment where people who work with or care for athletic horses are likely to use similar terminology.

The fact that observers were able to judge the horses’ emotional expressions based on 30 s videos indicates that our results may be relevant for the assessment of horse-human interactions, in which an immediate assessment of the horse’s emotional expressivity is essential. The present study demonstrated agreement with previous studies on QBA in other animal species in finding mostly good interobserver reliability for a fixed list of QBA terms applied to pre-or post-race video-based assessments of horses. Using the FCP methodology, more than 16 observers showed consensus, both statistically and semantically, in their blinded judgments of horses’ emotional expressions in the competitive environment. This evaluation could be a promising tool to promote better physical and mental conditions and welfare of racehorses and other animal species because it meets the criteria (human-animal relationship, comfort behavior, and social behavior) described for the correct evaluation of animal behavior (21). From a sport horse perspective, the proposed tool may be useful to support the social license to operate of the horse race worldwide.

A limitation of this study was some effects that may influence the behavior of horses in the race environment. The groom’s influence, the use of bit and earplugs and the moment of the assessment were not controlled in the current study, and it possible have influenced the horse behavioral response in the video. However, we understand that all these components are part of the race environment in a real scenario. Future studies could test the proposed tool in different race scenarios around the world.

## 5. Conclusion

The present study was effective in creating a fixed list of seven descriptors (agitated, troubled, restless, irritating, focused, relaxed, and peaceful) that could be used to assess the emotional expressivity of horses in a racing environment. The validation and application of qualitative behavioral assessment (QBA) terms through the Free Choice Profiling process and content validation allowed access to the terms that best characterized the emotional expressivity of the horses, aiming at better physical and mental conditions and welfare of these animals before and after races. Further studies can use this list of descriptors to correlate it with performance and welfare of racehorses.

## Data availability statement

The original contributions presented in the study are included in the article/Supplementary material, further inquiries can be directed to the corresponding author.

## Ethics statement

This study was approved by the Committee on Ethics in Animal Use (#8656240521) of the School of Veterinary Medicine and Animal Science, University of São Paulo, Brazil.

## Author contributions

RB and TO: conceptualization, acquisition of financing, and supervision. FJ and PS: data curation. FJ, PS, PT, RB, and TO: methodology. FJ and TO: project administration. PS: software. FJ: writing—original draft. RB, TO, and PS: writing—proofreading and editing. All authors contributed to the article and approved the submitted version.

## Conflict of interest

The authors declare that the research was conducted in the absence of any commercial or financial relationships that could be construed as a potential conflict of interest.

## Publisher’s note

All claims expressed in this article are solely those of the authors and do not necessarily represent those of their affiliated organizations, or those of the publisher, the editors and the reviewers. Any product that may be evaluated in this article, or claim that may be made by its manufacturer, is not guaranteed or endorsed by the publisher.
