# Supplementary material for: Development of a fixed list of descriptors for the qualitative behavioral assessment of thoroughbred horses in the racing environment
Source: Front Vet Sci. 2023 Aug 10;10:1189846. doi: 10.3389/fvets.2023.1189846 (PMC10482396; doi:10.3389/fvets.2023.1189846)
Supplement: Supplementary file 1 [file Data_Sheet_1.PDF]

Supplementary table: showing descriptors in Portuguese and English.

**Descritores**	**Descriptors**
Agitado	Agitate
Alerta	Alert
Apatico	Apathetic
Atento	Attentive
Ativo	Active
Calmo	Calm
Concentrado	Concentrated
Curioso	Curious
Desconfiado	Suspicious
Focado	Focused
Incomodado	Troubled
Inquieto	Restless
Irritado	Irritated
Relaxado	Relaxed
Tenso	Tense
Tranquilo	Peaceful
